# A Regional Survey on Merkel Cell Carcinoma: A Plea for Uniform Patient Journey Modeling and Diagnostic–Therapeutic Pathway

**DOI:** 10.3390/curroncol29100570

**Published:** 2022-09-30

**Authors:** Michela Roberto, Andrea Botticelli, Alessio Caggiati, Alberto Chiriatti, Carlo Della Rocca, Virginia Ferraresi, Felice Musicco, Giovanni Pellacani, Paolo Marchetti

**Affiliations:** 1 Medical Oncology Unit A, Umberto I University Hospital, Sapienza University, 00100 Rome, Italy; 2 Department of Radiological, Oncological and Anatomopathological Science, Umberto I University Hospital, 00161 Rome, Italy; 3 Istituto Dermopatico dell’Immacolata IRCCS, 00167 Rome, Italy; 4 Primary Care Physician Azienda Sanitaria Locale (ASL) Roma 3, 00125 Rome, Italy; 5 Department of Medico-Surgical Sciences and Biotechnologies, Sapienza University, Pathology Service, Umberto I University Hospital, 00185 Rome, Italy; 6 Sarcomas and Rare Tumors Departmental Unit, IRCCS Regina Elena National Cancer Institute, 00144 Rome, Italy; 7 IRCCS IFO Regina Elena and San Gallicano Institute, 00144 Rome, Italy; 8 Dermatology Clinic, Department of Clinical, Internal, Anesthesiological and Cardiovascular Science, University of Rome, 00185 La Sapienza, Italy; 9 Oncology Unit, Department of Clinical and Molecular Medicine, Sapienza University, Sant’Andrea Hospital, 00187 Rome, Italy

**Keywords:** Merkel cell carcinoma (MCC), diagnostic and therapeutic pathway, survey, avelumab, multidisciplinary team (MDT)

## Abstract

Merkel cell carcinoma (MCC) is a rare and aggressive cutaneous neuroendocrine cancer that usually affects the elderly and immunosuppressed in sun-exposed areas. Due to its rarity, it is frequently unrecognized, and its management is not standardized across medical centers, despite the more recent availability of immunotherapy, with avelumab as first-line treatment improving the prognosis even in advanced stages of disease. We conducted a purpose-designed survey of a selected sample of physicians working in the Lazio region, in Italy, to assess their awareness and knowledge of MCC as well as their perspective on assisted diagnostic and therapeutic pathways. The Lazio region, and in particular Rome, is one of the most important academic and non- academic center in Italy dedicated to the diagnosis and treatment of skin cancer. A total of 368 doctors (including 100 general practitioners, 72 oncologists, 87 dermatologists, 59 surgeons, and 50 anatomopathologists) agreed to be part of this survey. Surgeons, oncologists, and dermatologists thought themselves significantly more updated on MCC than primary care physicians, but more than half of the interviewees are interested in CCM training courses and training with clearer and more standardized care pathways. Significant differences have been reported from survey participants in terms of multidisciplinary team set up for MCC management. The identification of specialized centers and the improvement of communication pathways among different specialties, as well as between patients and physicians, could be very beneficial in improving patients’ journey modeling and starting a uniform diagnostic and therapeutic pathway for MCC patients in the new era of immunotherapies.

## 1. Introduction

Merkel cell carcinoma (MCC) is a rare and highly aggressive cutaneous neuroendocrine carcinoma that was first described as “trabecular carcinoma” in 1972 [1]. The immunophenotype of MCC cells have been found to be different from the progeny of mature normal Merkel cells (Cytokeratin 20—CK20+), and thus the true origin of MCC remains unknown [2].

MCC mainly affects elderly men with fair skin who develop immunosuppression due to organ transplant or HIV infection [3]. MCC is related to chronic UV exposure and/or to the Merkel cell polyomavirus (MCPyV) infection, which is reported in more than 80% of cases within MCC in Western countries [4]. Although the diagnosis of MCC has markedly increased, most likely due to the growing elderly population as well as the improved recognition of the MCC lesion [5], its incidence rate is estimated to represent less than 1% of all cutaneous malignancies in Europe [6], and the incidence of MCC increases proportionally with age ranging from 0.1 per 100,000 in young people to 9.8 in the elderly [6,7].

MCC occurs as an irregular erythematous nodule, isolated and rapidly growing within the skin layer, which is diagnosed through clinical examination followed by tissue biopsy, and demonstrates typical histopathological neuroendocrine features [8]. 

Prognosis depends on disease staging and treatment efficacy. As it is characterized by rapid local growth, MCC tends to metastasize to the lymph nodes and distant organs, resulting in very poor prognosis [9]. At the time of diagnosis, approximately 50–65% of patients present with localized disease, 25–50% with regional metastasis, and 10%, with distant metastasis [10]. The 5-year survival is 50–60% for localized disease, while for lymph-nodes and distant metastasis, it is 30–35% and 14%, respectively. Before the immunotherapy era, in advanced MCC, cytotoxic chemotherapeutics were usually adopted even if responses were rarely durable and high toxicity was observed [11]. Currently, chemotherapeutic regimens have been overcome with immunotherapy or only used as palliative strategies. Immunotherapy has profoundly changed the prognosis of MCC patients, even in advanced stages. Avelumab is a monoclonal antibody that binds to the ligand of programmed death-1 receptor (PD-L1), administered at a dose of 10 mg/kg every 2 weeks in patients with metastatic disease, who are considered unsuitable for locoregional treatment or for patients who relapse after surgery and/or radiotherapy. The single-arm, phase 2 trial (JAVELIN Merkel 200) investigated avelumab monotherapy in patients with metastatic MCC in two cohorts: second-line or later treatment in patients who had disease progression after one or more lines of chemotherapy (Part A); and first-line treatment (Part B) [12]. The latest published data in the enrolled 88 patients with stage IV chemotherapy-refractory (Part A), histologically confirmed MCC, reported an ORR of 33.0% (95% CI 23.3–43.8%), including a complete response in the 11.4% (10 patients), with a median duration of response of 40.5 months [13]. With a median follow-up of more than 44 months, the median OS was 12.6 months (95% CI 7.5–17.1 months). Furthermore, a 5-year OS rate of 26% (95% CI 17–36%) was recently reported [14,15].

Based on the Javelin 200 study, in 2017, the European Medicines Agency approved avelumab as monotherapy for the treatment of metastatic MCC regardless of the line of therapy, and in Italy, avelumab received reimbursement from the Italian Medicines Agency (Agenzia Italiana del Farmaco, AIFA, Roma, Italy) in 2018.

Currently, other immunotherapies, such as Nivolumab and Pembrolizumab, are not indicated for MCC in Italy [16,17].

Considering on the one hand its rarity and its unspecific clinical manifestations, MCC is still an incompletely acknowledged disease, and on the other hand, the better chance of cure with immunotherapy, the improvement of its detection and early diagnosis as well as the beginning of the treatment by an expert multidisciplinary team discussion (MTD) are of great importance for patient outcome [18]. The complexity of the disease and multiple comorbidities affecting most patients require the implementation of a territorial network, including primary care.

The Italian National Healthcare Service (NHS) is a public system essentially organized on a regional basis. Therefore, we conducted a survey on a selected sample of primary care physicians and specialists involved in the management of cutaneous tumors in the Lazio region, where specialized centers, especially in Rome, are also a national reference point. The purpose of this investigation was to assess the knowledge of MCC among physicians, and their point of view regarding the elaboration of a shared regional diagnostic and therapeutic pathway.

Moreover, the participants’ specific interest in training courses was requested to improve the patient journey modeling.

Finally, based on the literature data and participants support, we reported a clinical step-by-step process (patient journey modeling) with a multidisciplinary approach to enhance the management of patients affected by MCC and to propose an example of international assisted diagnostic and therapeutic pathways.

## 2. Materials and Methods

### 2.1. Survey Design

This study was a professional survey of physicians involved in the management of skin tumors in Italy, in the Lazio region, since many academic and non-academic major hospitals are located in Rome. These centers also attract patients with rare skin tumors from other Italian regions and are selected among the most important national centers.

This survey was elaborated by a multidisciplinary scientific board composed of three oncologists, one dermatologist, one plastic surgeon, one hospital pharmacist, one anatomopathologist, and one general practitioner. All board members are recognized as key opinion leaders in skin cancer management and have an academic reputation or belong to national and international scientific societies.

The survey was conducted using a computer-assisted web interviewing (CAWI) system according to other studies [19]. The contact information for eligible physicians was extracted from the database of the National Sanitary Service or the scientific societies. 

The survey was active for three weeks, in June 2021, and submitted to a panel of physicians via e-mail, using a password-protected web link. All survey responses were anonymized and handled via remote dispersed geographic participation among a sample of more than 500 physicians located in the Lazio region. 

The invitation to the survey declared the content of the study and provided the opportunity to join the project, deny involvement, or terminate participation at any time without penalty. Informed consent was obtained from all participants through an information sheet, before starting the survey (Dlgs. n. 196/2003—Regolamento Europeo (EU) 679/2016).

The questions were submitted as an anonymous branching survey that utilized skip patterns to ensure that respondents only replied to the questions customized to their profile. Responders were divided into three groups: (i) general practitioners (GPs); including primary care physicians; (ii) specialists involved in cutaneous tumor management, including oncologists, dermatologists, surgeons, or plastic surgeons; and (iii) anatomopathologists.

The survey took an average of 15–20 min to complete and was purpose-designed to meet the objectives of the survey, relating to the following main areas: (1) awareness and multidisciplinary approach towards MCC management; (2) attitude and approach of the clinicians towards MCC diagnosis; (3) general management of therapy and follow-up; and (4) consideration regarding the importance of regional diagnostic and therapeutic pathways -related training courses.

Since this was the first initiative in this area, the questions selected for this survey were not externally validated. Due to the non-experimental human research nature of this study, ethical review and approval were not required. Thus, ethical approval was waived by the ethics committee of Sapienza University in Rome.

All methods were performed in accordance with relevant guidelines and regulations.

### 2.2. Statistical Analysis 

Question responses were either multiple-choice or a single answer according to the nature of the question and were tabulated by medical specialty or aggregated and summarized descriptively by frequency and percentage.

The collected data were processed to investigate the responses of the individual subgroups interviewed (Supplementary Tables S1–S3) and comparative analyses were conducted between the different subgroups belonging to the sample to highlight the results that emerged through the application of modeling filters. The description of the data also included proportions and frequencies. Descriptive statistics were used to summarize the participants’ demographic characteristics and their answers to the survey questions. If necessary for statistical analysis purposes, the scores obtained from the answers were grouped into three possible conditions: (i) 0, 1, 2, and 3 (meaning “not sufficiently”); (ii) 4, 5, 6, and 7 (meaning “fairly”); (iii) 8, 9, and 10 (meaning “well”). Pearson’s chi-squared test was carried out to measure the association(s) between the answers to the survey and the different categories of physicians. For all analyses, a *p* value < 0.05 was considered statistically significant. Data were analyzed using SPSS, version 25.0 (IBM SPSS, Chicago, IL, USA).

## 3. Results

### 3.1. Participant Characteristics

A total of 368 physicians accepted to participate.

Professional categories included primary care physicians (100 respondents), oncologists (72 respondents), dermatologists (87 respondents), surgeons/plastic surgeons with expertise in cutaneous tumor management (59 respondents), anatomopathologists (50 respondents), and the scientific board (8 respondents) (Supplementary materials). The survey participants areas are mainly Rome and Latina, where the concentration of centers of expertise is significant.

The GPs and the dermatologists were both significantly younger than the other participants (Table 1).

### 3.2. Awareness and Understanding of Merkel Cell Carcinoma

Primary care physicians play an important role in the early detection of skin tumors, and both patient and physician awareness regarding the signs and symptoms of early MCC remains crucial. The analysis of the awareness about the MCC revealed that 50% of the primary care physicians considered their training in cutaneous tumors fairly adequate, and 58% of respondents claimed to be familiar with MMC. However, 72% of primary care physicians considered themselves not sufficiently updated on the MCC and almost all would be willing to attend specific training courses on rare forms of skin cancer. 

Compared with primary care physicians, specialists involved in cutaneous tumor management are significantly more updated on MCC. Specifically, 74% of surgeons/plastic surgeons reported that they are well updated about MCC, versus 44 % of oncologists, 44% of dermatologists, 16% of anatomopathologists, and 0% of primary care doctors (Table 2).

Between 40 and 50% of GPs fully agree that a specific training course in rare cutaneous tumors would be beneficial for their practice (Table 2).

Regarding MCC care pathways, only half of the GPs interviewed (55%) knew specific MCC care centers in the Lazio region; they were aware of the main centers for the management of cutaneous tumors (92%) or oncologic academic centers (8%). However, only 14% answered that they were in direct contact with expert physicians in specialized MCC care centers.

Most participants were interested in developing a specific and standardized care pathway for people with MCC.

In detail, 58% of GPs, 63% of the oncologists, 71% of the dermatologists, 86% of the surgeons/plastic surgeons, and 54% of the anatomopathologists would like to be informed about innovative diagnostic and therapeutic pathways for patients affected by MCC in their region (Table 2).

Compared with other specialists, the anatomopathologists included in this survey were older and considered themselves updated, however, like other participants, they considered that diagnostic and therapeutic pathways about MCC in the Lazio Region would be very useful in reducing the risk of bias. Moreover, they would like to participate in specific training programs, even if they are not essential to their daily work (Table 2).

**Figure 1:**
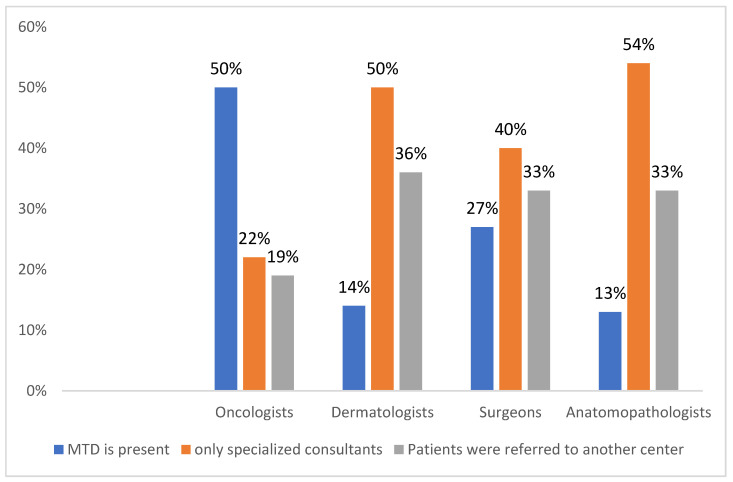
Multidisciplinary team discussion (MTD) involved in MCC management in the working center of each interviewed physician.

The effective management of MCC requires multidisciplinary care to optimize patient outcomes [20]; however, the availability of an MDT including several specialized physicians is reported to be significantly different in the working centers of each interviewed doctor: 50%, 14%, 27%, and 13% of oncologists, dermatologists, surgeons, and anatomopathologists, respectively. (*p* < 0.0001) (Figure 1).

**Figure 2:**
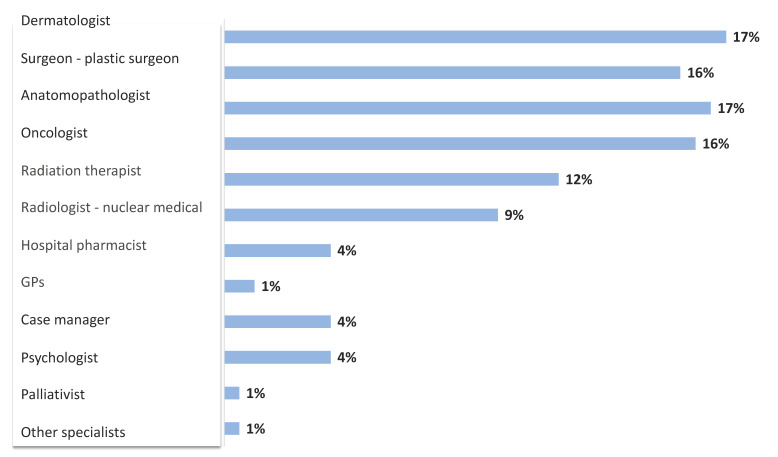
Specialists involved in multidisciplinary MCC management team according to participants’ answers.

The multidisciplinary team usually includes a dermatologist, a surgeon or plastic surgeon, a medical oncologist, and an anatomopathologist; less frequently, the involvement of a radiation oncologist, a radiotherapist, a nuclear medicine physician, and a psychologist may be reported, despite the substantial psychological impact of MCC on patients and families [21]. Additional specialistic services are required for patients with specific comorbidities. Although the presence of a case manager in charge of planning the procedures scheduled for the patient is recommended, the specific designation of this fundamental professional figure in the multidisciplinary team is still uncommon and is mainly reported by oncologists. (Figure 2).

**Figure 3:**
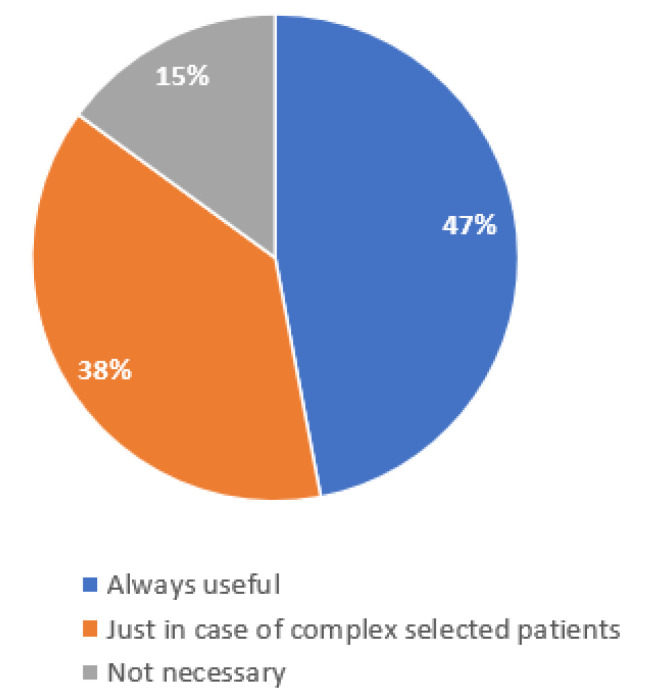
The orientation of participants in the use of telemedicine to encourage interaction among MDT members.

Most of the interviewed physicians are interested in enhancing the multidisciplinary management of MCC patients, as well as in using telemedicine support, referring to a specialized team center [22]; 38%, however, believed that telemedicine could only be useful in the management of more complex cases. (Figure 3).

### 3.3. Attitude and Approach of the Clinicians towards MCC Diagnosis

Specialists were asked how many MCC patients were treated per year: all the interviewed oncologists answered that they diagnosed at least one case of MCC per year at their center. Only 6% of the oncologists answered an average of “more than five cases”. However, 7% of the dermatologists, 13% of surgeons, and 10% of the anatomopathologists declared they had not found any case of MCC over a 12-month period.

The presence of a hard grey-pink to violet lump of skin lesion in UV-exposed body areas, which grows rapidly, mainly in elderly patients, suggests a neodiagnosis of MCC.

Regarding the main risk factors for MCC, 69% of the oncologists reported tumor dimensions (T > 2 cm), while 86% of the dermatologists and 80% of the surgeons answered both chronic immunocompromised state/transplant/HIV/ LCC and lymphovascular invasion.

Among the GPs, 40% declared that they suspected MCC in the presence of a blue-red lesion, in sun-exposed areas of the body, in elderly and/or chronically immunocompromised patients. Seventy-four percent of them referred the patient to another specialist (dermatologist or surgeon and plastic surgeon) or to a specialized center.

Among dermatologists, 64% considered dermatoscopy as a useful tool in the differential diagnosis of MCC.

Five cases per year were diagnosed by 14% of anatomopathologists. Half of them considered that the histological diagnosis of MCC does not require ultra-specialistic expertise. Moreover, 38% of the anatomopathologists did not refer the patients to other colleagues and no one was asked to revise previously examined histological material. In addition, 68% of the anatomopathologists stated that they did not carry out the search for infection by Mpcyv.

Almost all the specialists (100% of oncologists, 86% of dermatologists, and 80% of surgeons) recommend the biopsy of sentinel lymph nodes.

In terms of diagnostic staging, there were some differences among specialists. Indeed, asking them which primary imaging test is necessary for the disease staging, 25% of the oncologists answered total body CT scan or 18-FDG-PET plus brain CT scan/MRI; 26% of the dermatologists recommended total body CT scan or abdominal/pelvic and lymph-nodes ultrasound; while 40% of surgeons would refer the patients to a specialized center for the MCC staging.

### 3.4. General Management of MCC: Treatment and Follow-Up

Surgery is generally considered the first-line treatment for patients with local or regional diseases [23]. Fifty percent of the dermatologists and 60% of the surgeons/plastic surgeons answered that, in cases of suspected MCC, they removed the entire lesion with narrow margins of healthy skin and eventually, after waiting for the definitive histology, performed a flap or skin graft. In terms of surgical treatment, dermatologists and surgeons reported a different approach: 57% of the dermatologists undertook the complete surgical removal of an MCC on the primitive lesion, while 53% of the surgeons undertook the complete surgical removal following a wide excision of the MCC lesion; a 1 cm margin and 2 cm margin as the minimum margin of excision from the primitive lesion, were recommended by 43% and 29% of the dermatologists, respectively.

**Figure 4:**
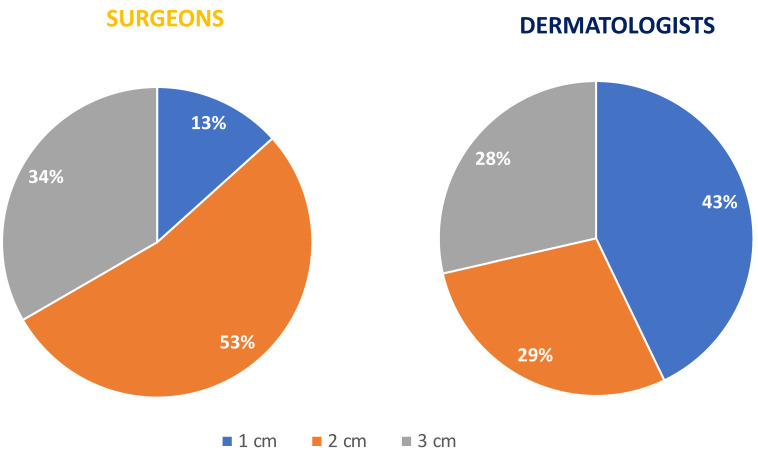
Plastic surgeons and dermatologists’ approaches to the minimum margin of excision from the primitive lesion.

A 1 cm margin is recommended by 13% of the surgeons and a 2 cm margin by 53% of the surgeons/plastic surgeons. (Figure 4).

**Figure 5:**
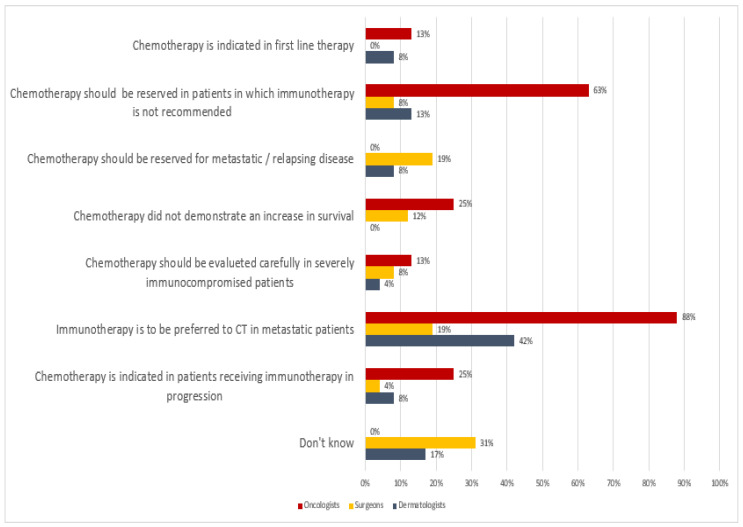
Orientation of participants to MCC first-line oncological treatment.

Regarding the choice of first-line oncological treatment, the oncologists responded that immunotherapy is the preferred choice for first-line treatment in metastatic patients (88%), and they only used chemotherapy in patients with contraindications to immunotherapy (63%); dermatologists preferred immunotherapy to chemotherapy in an advanced disease stage (42%); whereas most of the surgeons (31%) answered “I don’t know”. The main reason is linked to the fact that Italian surgeons and plastic surgeons are not allowed to prescribe any systemic treatment to cancer patients. In most cases, oncologists and dermatologists are the only ones authorized to prescribe systemic treatment (Figure 5).

With regard to immunotherapy, 81% of the oncologists indicated avelumab as the first choice in advanced MCC, except for those patients having undergone organ transplant receiving immunosuppressive therapy (50%), or patients with autoimmune disease during active phase (31%). However, 50% of the oncologists reported that there were no absolute contraindications for immunotherapy.

**Figure 6:**
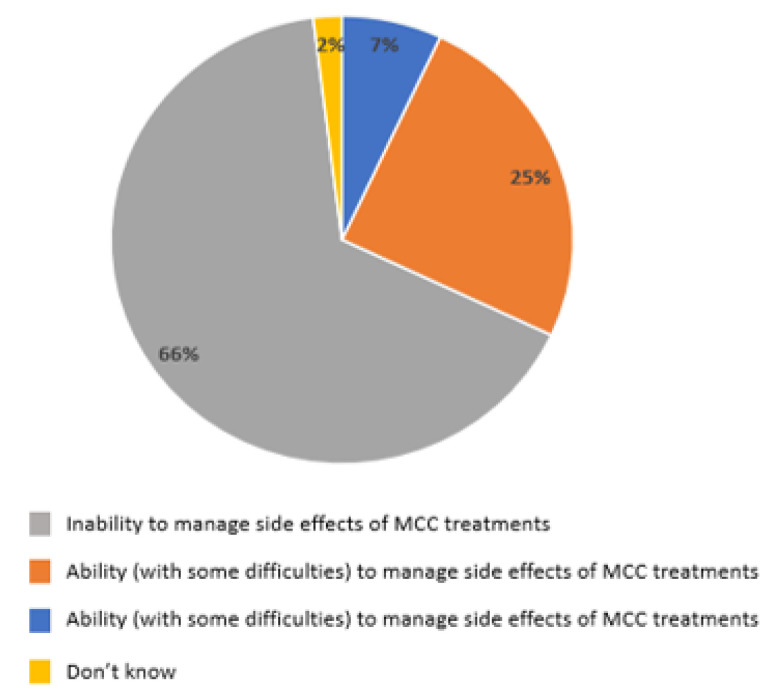
GPs ability to manage the side effects of chemotherapy and immunotherapy administered for the treatment of MCC.

When asked about the management of their patients receiving oncological treatments, 66% of GPs considered themselves unable to manage the side effects of the MCC treatments, 25% answered they were able but with some difficulties, and only 7% were capable (Figure 6).

**Figure 7:**
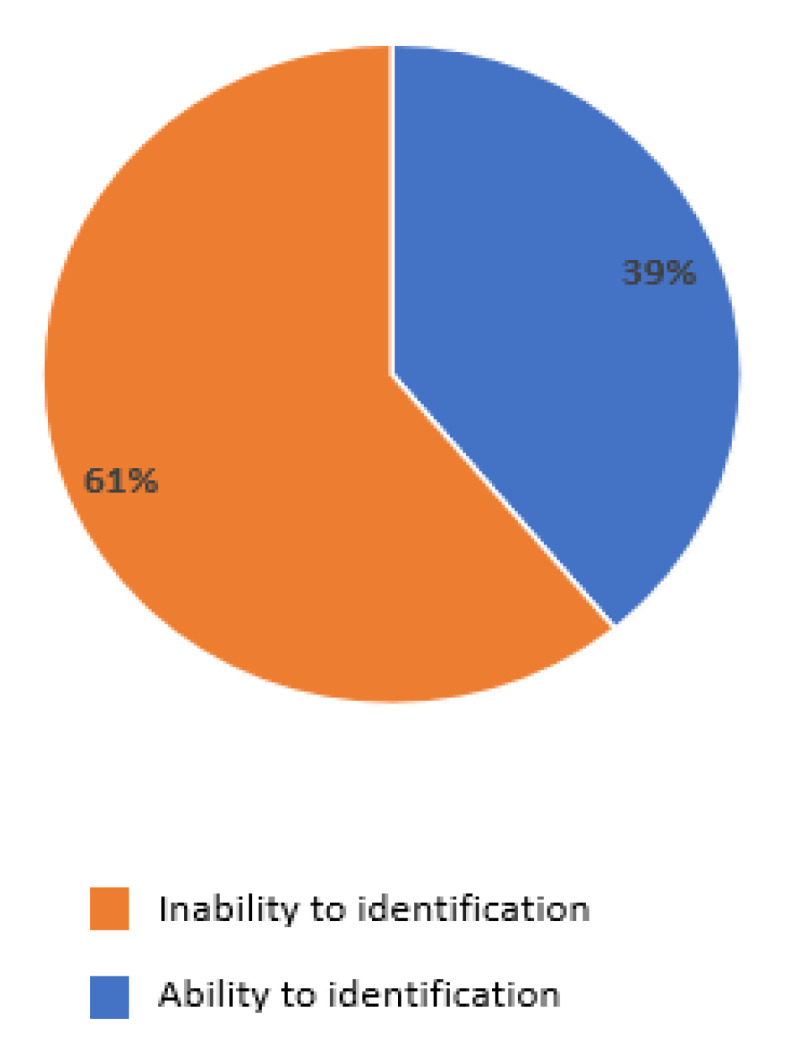
Identification of the referring specialists’ or reference center by GPs, in the case of side effects of MCC treatments.

Moreover, 61% answered that they did not know which specialist referred the patients in the case of side effects, and were not in direct contact with the specialists’ reference center (Figure 7).

On the other hand, 49% of GPs answered that they were part of an efficient palliative care network.

**Figure 8:**
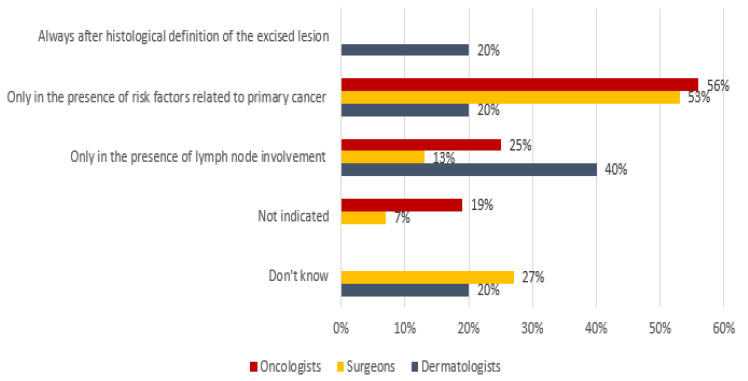
Orientation of participants to radiotherapy treatment for MCC.

Regarding radiotherapy, clear evidence from the literature is lacking [24,25,26,27]. Among the oncologists and surgeons/plastic surgeons, 56% and 53% indicated radiotherapy in the presence of risk factors related to the primitive tumor, respectively, whereas 40% of the dermatologists only considered it in the presence of lymph node involvement (Figure 8).

**Figure 9:**
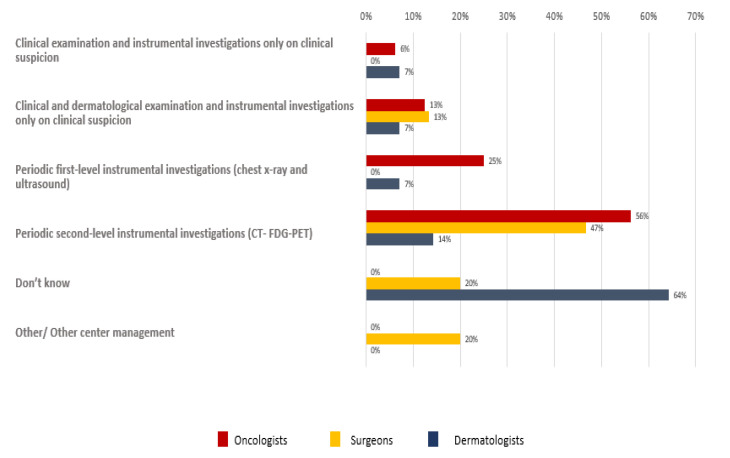
Orientation of participants to follow-up program for patients with resected MCC diagnosis.

As regards the follow-up program, different results were reported among different participants (Figure 9).

**Figure 10:**
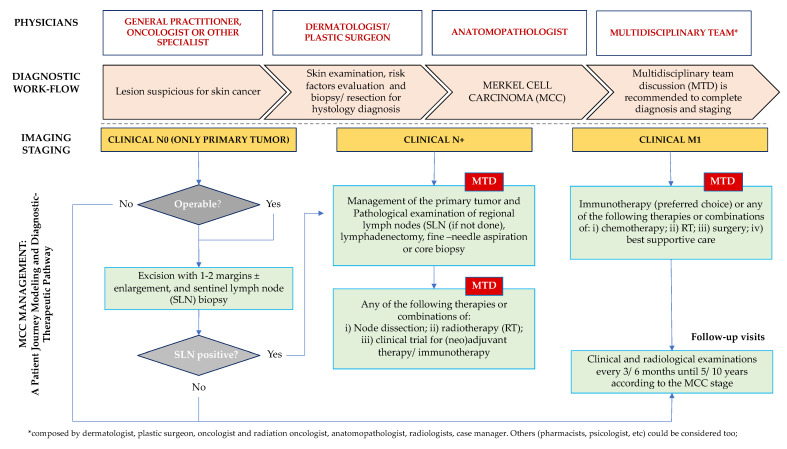
Road map of a patient journey modeling and diagnostic–therapeutic pathway.

Based on the aforementioned data and survey participants’ contributions, a step-by-step clinical process (patient journey modeling) with a multidisciplinary approach is presented below with the aim of enhancing the management of patients affected by MCC and to propose an example of international diagnostic and therapeutic pathway (Figure 10).

## 4. Discussion

This was the first survey on MCC perceptions, awareness, and knowledge among physicians of different medical specialties that are active in the Lazio area, particularly in Rome, which is one of the most important areas for academic and non-academic centers in Italy for the diagnosis and treatment of skin cancer. Based on very few new cases per year, both GPs and specialists highlight the need for a specialized training course on MCC and a shared regional diagnostic and therapeutic pathway for patients with MCC, such as the one already set up in the Veneto area [17]. Accordingly, in this study, we presented a proposal for patient journey modeling and a diagnostic–therapeutic pathway.

The involvement of GPs was considered fundamental to the early diagnosis of MCC and adequate local follow-up for patients treated in specialized centers. GPs, during their daily practice, face a wide range of pathologies and it is difficult for them to have the appropriate knowledge and skills for rare diseases. A fair percentage of GPs referred the patient to a specialist for diagnostic confirmation. It is also interesting that most of them are keen to have a standardized diagnostic and therapeutic pathway to follow.

In these centers, the availability of a multidisciplinary team, possibly including different specialists from different areas of competence, would help the patient journey modeling and the management of this rare disease.

Regarding the recognition of MCC risk factors, oncologists, surgeons/plastic surgeons, and dermatologists were able to detect the principal signs of a skin lesion suspected to be MCC.

Surgical treatment remains the preferred first step in the management and treatment of localized disease, with the purpose of widening the excision to a negative histological margin. There is non-consensus in the literature on the ideal lateral margin and some biases, common to other international settings, persist about surgical treatment in terms of surgical margins and sentinel lymph node removal. Indeed, the sentinel lymph node biopsy is highly variable and it is mainly driven by a primitive lesion’s location, the age of the patient, comorbidities, and other factors that would affect the skin, such as the thin scalp of the elderly. In this survey, surgical excision with 1 cm and 2 cm margins was recommended by 66% of the dermatologists and 72% of the surgeons/plastic surgeons, respectively.

Finally, although the histological diagnosis of MCC is technically simple, a second opinion may be asked, such as for other rare tumors, in specialized centers, and to integrate the patient into specific treatment protocols as well as into a data program for further scientific studies.

Chemotherapy (cyclophosphamide, doxorubicin, epirubicin, and vincristine) is not indicated for the primary treatment of MCC and does not improve survival in patients with stage I–III disease. In the palliative setting, chemotherapy can be administered for metastatic disease, however these drugs are toxic and often not tolerated in elderly patients [28]. Preserving chemotherapy only in advanced disease and in patients that are not candidates for immunotherapy (immune-suppressive therapy, active HIV, HBV, or HCV infection) depends exclusively on the attitude of the oncologist, who plays a main role in the pharmacological management of MCC compared to other specialists.

The survey findings show that there is substantial agreement regarding the benefits of immunotherapy as first-line treatment for MCC. According to the product schedule, the recommended dose of avelumab as monotherapy is 800 mg, administered intravenously over 60 min every 2 weeks. The administration of avelumab should be continued according to the recommended schedule until disease progression or unacceptable toxicity. Patients must be premedicated with antihistamine and paracetamol prior to the first four infusions of avelumab. If the fourth infusion is completed without an infusion-related reaction, premedication for subsequent doses should be administered at the physician’s discretion. As with other checkpoint inhibitors, treatment modifications, dose escalation, or dose reduction are not recommended. Dosing delays or discontinuation may be required based on individual safety and tolerability. Certainly, including a hospital pharmacist in the MDT can be helpful in encouraging adherence to administration rules and reporting adverse events.

Only 7 of 100 GPs claim to be able to self-manage the side effects of oncological treatments. Because of a lack of connections between GPs and specialists, patients are forced to go directly to specialized centers and a need for a shared diagnostic and therapeutic pathways again arises to support GPs in interacting with specialists, even using telemedicine, to agree on how to manage the side effects of the treatments and reduce the number of hospital admissions. Furthermore, sharing contacts and information about the patient’s treatment after discharge would be very useful for GPs to manage the side effects of therapies and improve the quality of life of patients with MCC and their families.

There were different specialist approaches about radiotherapeutic treatment choice in this survey. Given the lack of a clear standard algorithm for adjuvant radiotherapy in MCC treatment [24,25,26], the inclusion of a radiation oncologist in the MDT is crucial. Finally, while there is a common sentiment in following personalized pathways designed on the basis of patient and disease characteristics, there are many contradictions about the follow-up process of MCC which may be due to the lack of standardized indications despite the international recommended guidelines [23]. Having local well-structured and widely recognized diagnostic and therapeutic pathways could improve the management of MCC follow-up.

## 5. Conclusions

Despite the well-known limitations of a cross-sectional purpose-designed survey, this study has important clinical implications. Given the heterogeneity of both diagnostic and therapeutic behavior, as well as the clinical need to standardize the medical definitions and indications that emerged from this survey, the aim of this project was to ensure that multidisciplinary and parallel skills such as dermatology, oncology, surgery, and anatomopathology converge in a transdisciplinary decision that brings cultural enrichment and better results. These specialties, together with other professionals such as pharmacists, radiation therapists, psychologists, and case managers, must work together to jointly define a uniform patient journey modeling that patients will follow to improve their outcomes.

It would be desirable to implement communication pathways between hospitals and territory health centers, not only about updated training courses, but also using the new digital technologies such as telemedicine to support GPs and other primary care professionals, despite their geolocation, in the management of patients affected by rare cutaneous tumors. This would translate into better disease management and the improved tolerability of treatments, compliance, and approach to the national guidelines, thus reporting a higher probability of being cared for and having longer survival rates among patients.

Finally, it is important to emphasize that, to improve the management of MCC, the early diagnosis and treatment are fundamental for the care of the patient, and the elaboration of shared knowledge is becoming increasingly necessary globally. Thus, with this survey, this study demonstrates that it is appropriate to organize a specific training course on MCC, as well as provide regional pathways shared by all physicians involved in the management of rare diseases including MCC.

These paths are well suited to the Italian Healthcare System, which is structured on a regional system but can also be exported to other countries’ models because it might avoid the bias of treatments, delineate new potential challenges for the teamwork involved in the management of MCC, and allow a better connection between GPs and specialized centers. Healthcare professionals ask to be constantly updated on the MCC care pathways available in their areas and to be able to work through telemedicine support. In addition, diagnostic and therapeutic pathways not only facilitate the referral of patients with MCC to specialized centers, but also, in cases of advanced and refractory disease, simplify the activation of palliative care services for fragile or end-of-life patients.

## Figures and Tables

**Table 1:** The distribution of epidemiological characteristics of all 368 physicians who participated in the survey, expressed as the number and percentages.

	General Practitioners	Oncologist	Dermatologist	Plastic Surgeon	Anatomopathologist
Total Number	100	72	87	59	50
**Age**					
<40	17 (17%)	18 (25%)	37 (43%)	8 (14%)	14 (28%)
41–60	55 (55%)	40 (56%)	43 (50%)	39 (66%)	23 (46%)
>60	28 (28%)	14 (19%)	7 (7%)	12 (20%)	13 (26%)
Median age	52.4	49.5	43.8	51.5	50.1
**Working place**					
Roma	95 (95%)	65 (90%)	83 (96%)	55 (93%)	45 (90%)
Latina	0	3 (4%)	2 (2%)	2 (3%)	4 (8%)
Fronsinone	0	2 (3%)	1 (1%)	1 (2%)	0
Viterbo	3 (3%)	2 (3%)	1 (1%)	1 (2%)	1 (2%)
Rieti	2 (2%)	0	0	0	0
**Sex**					
Female	37 (37%)	28 (39%)	33 (38%)	14 (24%)	22 (44%)
Male	63 (63%)	44 (61%)	54 (62%)	45 (76%)	28 (56%)

**Table 2:** Awareness and understanding of Merkel cell carcinoma among the 368 physicians who participated in the survey.

	General Practitioners N (%)	Oncologist N (%)	Dermatologist N (%)	Surgeons/Plastic Surgeons N (%)	Anatomopathologist N (%)
**Updating on MCC management**					
Not sufficiently	72 (72%)	14 (20%)	7 (8%)	8 (14%)	14 (28%)
Fairly	28 (28%)	26 (36%)	43 (49%)	39 (66%)	23 (46%)
Well	0 (0)	32 (44%)	37 (43%)	12 (20%)	13 (26%)
**The depiction of updating on regional** diagnostic and therapeutic pathways **for MCC**					
Not sufficiently	7 (7%)	0 (0)	0 (0)	0 (0)	6 (12%)
Fairly	35 (35%)	27 (37%)	25 (29%)	8 (14%)	17 (34%)
Well	58 (58%)	45 (63%)	62 (71%)	51 (86%)	27 (54%)

## Data Availability

The data used to support the findings of this study are available from the corresponding authors upon request.

## Supplementary Materials

The following supporting information can be downloaded at: https://www.mdpi.com/article/10.3390/curroncol29100570/s1, Table S1: Questionnaire items with answers of 100 General Medicine doctors, expressed as percentage, Table S2: Questionnaire items with answers of specialized physicians involved in cutaneous tumor management, expressed as percentages, Table S3: Questionnaire items with answers (expressed as percentages) of 50 Anatomo-pathologists involved in cutaneous tumor management.

## References

[1] Toker C. (1972). Trabecular Carcinoma of the Skin. Arch. Dermatol..

[2] Liu W., MacDonald M., You J. (2016). Merkel cell polyomavirus infection and Merkel cell carcinoma. Curr. Opin. Virol..

[3] Lanoy E., Costagliola D., Engels E.A. (2010). Skin cancers associated with HIV infection and solid-organ transplantation among elderly adults. Int. J. Cancer.

[4] Becker J.C., Houben R., Ugurel S., Trefzer U., Pföhler C., Schrama D. (2009). MC polyomavirus is frequently present in Merkel cell carcinoma of European patients. J. Investig. Dermatol..

[5] Walsh N.M., Cerroni L. (2021). Merkel cell carcinoma: A review. J. Cutan. Pathol..

[6] Becker J.C., Stang A., Hausen A.Z., Fischer N., DeCaprio J.A., Tothill R.W., Lyngaa R., Hansen U.K., Ritter C., Nghiem P. (2017). Epidemiology, biology and therapy of Merkel cell carcinoma: Conclusions from the EU project IMMOMEC. Cancer Immunol. Immunother..

[7] Paulson K.G., Park S.Y., Vandeven N.A., Lachance K., Thomas H., Chapuis A.G., Harms K.L., Thompson J.A., Bhatia S., Stang A. (2018). Merkel cell carcinoma: Current US incidence and projected increases based on changing demographics. J. Am. Acad. Dermatol..

[8] Patel P., Hussain K. (2021). Merkel cell carcinoma. Clin. Exp. Dermatol..

[9] Zwijnenburg E.M., Lubeek S.F.K., Werner J.E.M., Amir A.L., Weijs W.L.J., Takes R.P., Pegge S.A.H., van Herpen C.M.L., Adema G.J., Kaanders J.H.A.M. (2021). Merkel Cell Carcinoma: New Trends. Cancers.

[10] Iyer J.G., Storer B.E., Paulson K.G., Lemos B., Phillips J.L., Bichakjian C.K., Zeitouni N., Gershenwald J.E., Sondak V., Otley C.C. (2014). Relationships among primary tumor size, number of involved nodes, and survival for 8044 cases of Merkel cell carcinoma. J. Am. Acad. Dermatol..

[11] Nghiem P., Kaufman H.L., Bharmal M., Mahnke L., Phatak H., Becker J.C. (2017). Systematic literature review of efficacy, safety and tolerability outcomes of chemotherapy regimens in patients with metastatic Merkel cell carcinoma. Future Oncol..

[12] Kaufman B., Shapira-Frommer R., Schmutzler R.K., Audeh M.W., Friedlander M., Balmaña J., Mitchell G., Fried G., Stemmer S.M., Hubert A. (2015). Olaparib monotherapy in patients with advanced cancer and a germline BRCA1/2 mutation. J. Clin. Oncol..

[13] D’Angelo S.P., Bhatia S., Brohl A.S., Hamid O., Mehnert J.M., Terheyden P., Shih K.C., Brownell I., Lebbé C., Lewis K.D. (2020). Avelumab in patients with previously treated metastatic Merkel cell carcinoma: Long-term data and biomarker analyses from the single-arm phase 2 JAVELIN Merkel 200 trial. J. Immunother. Cancer.

[14] D’Angelo S.P., Lebbé C., Mortier L., Brohl A.S., Fazio N., Grob J.J., Prinzi N., Hanna G.J., Hassel J.C., Kiecker F. (2021). First-line avelumab in a cohort of 116 patients with metastatic Merkel cell carcinoma (JAVELIN Merkel 200): Primary and biomarker analyses of a phase II study. J. Immunother. Cancer.

[15] D’Angelo S.P., Bhatia S., Brohl A.S., Hamid O., Mehnert J.M., Terheyden P., Shih K.C., Brownell I., Lebbé C., Lewis K.D. (2021). Avelumab in patients with previously treated metastatic Merkel cell carcinoma (JAVELIN Merkel 200): Updated overall survival data after >5 years of follow-up. ESMO Open..

[16] Topalian S.L., Bhatia S., Amin A., Kudchadkar R.R., Sharfman W.H., Lebbé C., Delord J.P., Dunn L.A., Shinohara M.M., Kulikauskas R. (2020). Neoadjuvant Nivolumab for Patients With Resectable Merkel Cell Carcinoma in the CheckMate 358 Trial. J. Clin. Oncol..

[17] Nghiem P.T., Bhatia S., Lipson E.J., Kudchadkar R.R., Miller N.J., Annamalai L., Berry S., Chartash E.K., Daud A., Fling S.P. (2016). PD-1 Blockade with Pembrolizumab in Advanced Merkel-Cell Carcinoma. N. Engl. J. Med..

[18] Rastrelli M., Del Fiore P., Buja A., Vecchiato A., Rossi C.R., Chiarion Sileni V., Tropea S., Russano F., Zorzi M., Spina R. (2020). A Therapeutic and Diagnostic Multidisciplinary Pathway for Merkel Cell Carcinoma Patients. Front. Oncol..

[19] Muscaritoli M., Corsaro E., Molfino A. (2021). Awareness of Cancer-Related Malnutrition and Its Management: Analysis of the Results From a Survey Conducted Among Medical Oncologists. Front. Oncol..

[20] Pillay B., Wootten A.C., Crowe H., Corcoran N., Tran B., Bowden P., Crowe J., Costello A.J. (2016). The impact of multidisciplinary team meetings on patient assessment, management and outcomes in oncology settings: A systematic review of the literature. Cancer Treat. Rev..

[21] Kaufman H.L., Dias Barbosa C., Guillemin I., Lambert J., Mahnke L., Bharmal M. (2018). Living with Merkel Cell Carcinoma (MCC): Development of a Conceptual Model of MCC Based on Patient Experiences. Patient.

[22] Aghdam M.R.F., Vodovnik A., Hameed R.A. (2019). Role of Telemedicine in Multidisciplinary Team Meetings. J. Pathol. Inform..

[23] Donigan J.M., Farma J.M., Fung M.K., Ghosh K., Grekin R.C., Harms K., Ho A.L., Holder A., Lukens J.N., Medina T. (2021). NCCN Guidelines Version 1.2021 Merkel Cell Carcinoma. https://www.nccn.org/login?ReturnURL=https://www.nccn.org/professionals/physician_gls/pdf/mcc.pdf.

[24] Petrelli F., Ghidini A., Torchio M., Prinzi N., Trevisan F., Dallera P., De Stefani A., Russo A., Vitali E., Bruschieri L. (2019). Adjuvant radiotherapy for Merkel cell carcinoma: A systematic review and meta-analysis. Radiother. Oncol..

[25] Gunaratne D.A., Howle J.R., Veness M.J. (2017). Definitive radiotherapy for Merkel cell carcinoma confers clinically meaningful in-field locoregional control: A review and analysis of the literature. J. Am. Acad. Dermatol..

[26] Hong A.M., Stretch J.R., Thompson J.F. (2021). Treatment of primary Merkel cell carcinoma: Radiotherapy can be an effective, less morbid alternative to surgery. Eur. J. Surg. Oncol..

[27] Zerini D., Patti F., Spada F., Fazio N., Pisa E., Pennacchioli E., Prestianni P., Cambria R., Pepa M., Grana C.M. (2021). Multidisciplinary team approach for Merkel cell carcinoma: The European Institute of Oncology experience with focus on radiotherapy. Tumori.

[28] Tello T.L., Coggshall K., Yom S.S., Yu S.S. (2018). Merkel cell carcinoma: An update and review: Current and future therapy. J. Am. Acad. Dermatol..

