# Supplementary material for: A Regional Survey on Merkel Cell Carcinoma: A Plea for Uniform Patient Journey Modeling and Diagnostic–Therapeutic Pathway
Source: Curr Oncol. 2022 Sep 30;29(10):7229–44. doi: 10.3390/curroncol29100570 (PMC9600681; doi:10.3390/curroncol29100570)
Supplement: Supplementary file 1 [file curroncol-29-00570-s001.zip › supplementary Tables_MCC_50722.pdf]

Table S1. Questionnaire items with answers of 100 General Medicine doctors, expressed as percentages

Q1. Working position	%
a) Primary care doctor	39
b) Training physician in general medicine	66
Q2. How old are you?	
a. <30 y	16
b. 30-40	29
c. 41-50	20
d. 51-60	18
e. >60	17
Q3. Which local area do you refer to?	
a. Roma1	35
b. Roma2	26
c. Roma3	39
d. Latina/Frosinone	0
Q4. In which province of Lazio do you work?	
a. Roma	95
b. Latina	0
c. Fronsinone	0
d. Viterbo	3
e. Rieti	2
Q5. Do you have a medical residency?	
a. Yes	39
b. Not	66
Q6. How much do you consider your knowledge on skin tumors is adequate ? (range 0-10)?	
a. 0	0
b. 1	8
c. 2	10
d. 3	20
e. 4	7
f. 5	22
g. 6	17
h. 7	5
i. 8	8
j. 9	2
k. 10	3
Q7. Do you know Merkel Cell Carcinoma (MCC)?	
a. Yes	58
b. Not	42
Q8. How much do you consider yourself updated on MCC?	
a) 0	13
b) 1	23
c) 2	20
d) 3	15
e) 4	10
f) 5	12
g) 6	3
h) 7	3
i) 8	0
j) 9	0
k) 10	0
Q9. How much importance would you give to a specific training about rare skin tumors in your everyday clinical practice?	
a. 0	0
b. 1	0

c. 2<br>d. 3<br>e. 4<br>f. 5<br>g. 6<br>h. 7<br>i. 8<br>j. 9<br>k. 10	3<br>5<br>5<br>10<br>9<br>17<br>16<br>13<br>22
Q10. How much importance would you give to a continous update about the diagnostic and therapeutic pathway of MCC in Lazio region?	
a. 0<br>b. 1<br>c. 2<br>d. 3<br>e. 4<br>f. 5<br>g. 6<br>h. 7<br>i. 8<br>j. 9<br>k. 10	0<br>0<br>5<br>2<br>5<br>10<br>7<br>13<br>15<br>13<br>30
Q11. Are you aware of specialized centers in your region for the management of skin cancers??	
a. Yes<br>b. Not	55 (IFO 51%, IDI 41%, Policlinico Umberto I 6%)<br>45
Q12. With regard to a skin lesion which are the elements that lead you to suspect a MCC diagnosis? (allowed multiple choices)	
a. Any lesion that increases volume over time<br>b. A rapidly growing skin lesion<br>c. A skin lesion that increases in volume, in photo-exposed areas<br>d. A skin lesion that grows rapidly and becomes symptomatic (itching, pain, bleeding)<br>e. A hard, gray-violet lesion on areas that are exposed in an elderly person<br>f. A duragree-violacelesion on areas that are chronically exposed (in an elderly person and/or)<br>g. g. I don't know	5<br>24<br>31<br>18<br>22<br>40<br>20
Q13. In the suspicion of a MCC, what is your orientation for the eventual removal?	
a. I always perform/I do a biopsy before the removal<br>b. Take-out/Take-out of the lesion keeping minimal margins<br>c. Take away/ I do take away of the lesion enlarging the margin and awainting histology before making a flap<br>d. Take away/ I do take away of the lesion widening margins and doing immediately unlembo<br>e. I leave the patient to other Specialist/Centre	7<br>2<br>14<br>3<br>74
Q14. Are you able to manage personally any side effects of cancer treatments in your patients with malignant skin tumors?	
a. Yes	7

b. Yes, with some difficulty<br>c. Not<br>d. I don't know	25<br>66<br>2
Q15. Do you know who to turn to, in case of side effects to oncological treatments in your patients with skin tumors?	
a. Yes<br>b. Not	39<br>61
Q16. If necessary, do you have any possibility of a direct contact with the specialist reference center?	
a. Yes<br>b. Not<br>c. I don't know	14<br>81<br>5
Q17. In the area where you work, are there defined paths of palliative care for cancer patients in advanced stage disease?	
a. Yes, there is an efficient palliative care network<br>b. Not<br>c. Yes, but only hospice center<br>d. Yes, but only home palliative care<br>e. I don't know	49<br>15<br>18<br>5

Table S2. Questionnaire items with answers of specialized physicians involved in cutaneous tumor management, expressed as percentages

	Oncologist (N= 72	Dermathologist (N= 87)	Surgeon (N= 59)
Q1. How old are you?			
f. <30	6	0	7
g. 30-40	19	43	7
h. 41-50	28	43	13
i. 51-60	28	7	53
j. >60	19	7	20
Q2. In which province of Lazio do you work?			
f. Roma		93	
g. Latina		2	
h. Frosinone		2	
i. Viterbo		2	
j. Rieti		0	
Q3. How much do you consider yourself updated on MCC? (range 0-10)?			
l. 0	0	0	0
m. 1	13	0	0
n. 2	6	0	0
o. 3	0	7	0
p. 4	6	21	0
q. 5	6	7	13
r. 6	19	14	7
s. 7	6	7	7
t. 8	25	21	27
u. 9	6	21	20
v. 10	13	0	27
Q4. How much importance would you give to a specific training about rare skin tumors in your everyday clinical practice?			
l) 0	0	0	0
m) 1	0	0	7
n) 2	0	0	0
o) 3	0	0	7

p) 4	0	0	13
q) 5	13	7	13
r) 6	25	29	7
s) 7	13	21	7
t) 8	25	21	13
u) 9	13	7	7
v) 10	13	14	27
Q5. How much importance would you give to a continous update about the diagnostic and therapeutic pathway of MCC in Lazio region?			
l. 0	0	0	0
m. 1	0	0	0
n. 2	0	0	0
o. 3	0	0	0
p. 4	6	7	0
q. 5	0	7	7
r. 6	19	7	7
s. 7	13	7	0
t. 8	19	21	33
u. 9	19	21	20
v. 10	25	29	33
Q6. In the center where you worki, how many Merkel cell carcinoma are averagely diagnosed in a year?			
l. 1 case	44	36	13
m. Up to 3 cases	31	14	27
n. Up to 5 cases	19	36	27
o. More than to 5 cases	6	7	20
p. No cases	0	7	13
q. I don't know	0	0	0
Q7. In your Center, is there a multidisciplinary evaluation for MCC?			
a. Yes, there is a dedicated multidisciplinary team	50	14	27
b. There isn't a multidisciplianary team, but there is an ad-hoc consulting	31	50	40
c. No, after the visit the patient is referred to another center	19	36	33
Q8. If there is the possibility of a multidisciplinary team evaluation, which specialists are involved in the process? (it's possible to flag all options)			
a. Dermatologists	17		
b. Plastic surgeons	16		
c. Anatomopathologists	17		
d. Medical oncologists	16		
e. Radiaton therapists	12		
f. Nuclear medicine physician	9		
g. Pharmacists	4		
h. General practitioners	1		
i. Case manager	4		
j. Psychologists	4		
k. Palliative care doctors	1		
Q9. Would it be necessary for you/your center to add a telemedicine platform to your multidisciplinary team to improve the management of MCC patients who agree to			

consult other specialists or specialized teams with an important experience on MCC?			
a. Yes, always<br>b. Yes, only for complex selected cases<br>c. No, the resources are available only in presence<br>d. No, I don't consider it necessary	47<br>38<br>11<br>9		
Q10. According to your knowledge and/or your experience, which patients do you consider at risk to have an MCC? (multiple answers are allowed, up to 3 answers. The numbers refer to the percentage of answers given to all answers)			
a. I don't know<br>b. Subjects with sun-damaged skin and high sun exposure	13<br>25	0<br>21	4<br>15
c. Poliomavirus virus positive subjects (MCPyV)	44	50	19
d. Genetic predisposition	0	0	0
e. Young aged subjects<br>f. Mainly subjects aged > a 50 years old	0<br>56	7<br>79	2<br>20
g. Immunocompromised patients<br>h. Dark skinned subjects	44<br>0	64<br>0	26<br>0
i. Bright skinned subjects	0	57	15
Q11. Which are the main three elements of a skin lesion which make you doubt of an MCC? (up to 3 answers allowed)			
a. I don't know<br>b. A grey-violet lesion in exposed areas of the body (elderly and/or chronically immunocompromised patients)		0<br>23	0<br>33
c. A grey-violet lesion in sun-exposed areas of the body of an elderly patient		20	7
d. A skin lesion that grows rapidly and becomes symptomatic (itchy, painful, bleeding)		13	22
e. A growing skin lesion in sun-exposed areas of the body		27	4
f. A skin lesion that grows rapidly		13	30
g. Any skin lesion that grows in time		3	46
Q12. Dermatoscopy in suspected MCC? (multiple answers allowed)			
a. It's not a suggested diagnostic tool<br>b. In selected cases it could be useful for differential diagnosis<br>c. It's always a fundamental diagnostic tool		0<br>64<br>36	
Q13. In a suspected MCC, which decision do you make about its removement?			
a. I refer the patient to a specialist or to a specialized center<br>b. I remove the lesion widening its margins and I immediately make a lembo.<br>c. I remove the lesion widening its margins and I wait for the histological results before making a lembo		29<br>14<br>50<br>7	7<br>60

d. I remove the lesion maintaining minimal margins			33
e. I always perform a biopsy before the removement			
Q14. The complete surgical removement of an MCC is usually performed:			
a. In a primitive lesion			
b. After an incisional biopsy		57	20
c. After an excisional biopsy		21	27
d. After a wided excisional biopsy		21	53
e. I refer the patient to another center			
Q15. Which width is recommended for the excision margins? (perilesional or around to the primitive lesion or to the scar), in case of previous excisional biopsy?			
a. 1 cm		43	13
b. 2 cm		29	53
c. 3 cm		28	34
Q16. In case of a histological diagnosis of an MCC do you recommend the sentinel lymph node biopsy?			
a. Yes	100	86	80
b. No	0	14	20
Q17. If present, which ones do you consider as risk factors? (multiple answers, all choices possible)			
a. T>2cm	69	64	80
b. Chronic immunocompromised state, transplant, HIV, CLL	44	64	80
c. Head-neck localization	31	57	60
d. Lymphovascular invasion	44	57	80
Q18. Which instrumental exams do you consider necessary for the staging of the disease? (multiple answers)			
a. I refer the patient to a specialized center		13	40
b. Total body FDG-PET scan + contrast brain CT/NMR only if di T> 2 cm (T2) or positive lymph nodes	19<br>13	13	33
c. Contrast total body CT only if T > 2 cm (T2) o positive lymph nodes	6	3	0
d. Total body FDG-PET+ brain contrast CT/NMR	25	13	33
e. Contrast total body CT	25	29	13
f. Only loco-regional lymph nodes ultrasound	0	0	0
g. Pelvic-abdominal and lymph nodes ultrasound	6	26	27
h. Chest X-ray	6	3	13
Q19. In which patients do you consider immunotherapy less indicated? (multiple answers allowed)			
a. Non-symptomatic HIV patiens in antiretroviral therapy	6		
b. AIDS patients	19		
c. Transplanted patients in immunosupressive treatment	50		
d. Patients with active autoimmune disease	31		

e. Patients with autoimmune disease in no immunosupressive treatment	25		
f. Patients with concomitant onco-hematologic disease in follow-up	0		
g. There is no absolute contraindication to immunotherapy	50		
Q20. When radiotherapy is indicated as MCC treatment?			
a. Always, after the histological definition of the removed lesion	0	20	0
b. Only in the presence of risk factors related to the primitive tumor	56	20	53
c. Only in the presence of lymphadenopathy	25	40	13
d. It's not indicated	19	20	7
e. I don't know	0	20	27
Q21. About the oncological treatment of MCC, according to you? (multiple choices):			
a. Chemotherapy is indicated as first line treatment	13	8	0
b. Chemotherapy is indicated only in patients unfitted to immunotherapy			
c. Chemotherapy is indicated in metastatic disease/relapse	63	13	8
d. Chemotherapy hasn't shown improvement in overall survival	0	8	19
e. Chemotherapy shoud be carefully valuated in immunocompromised patients	25	0	12
f. Immunotherapy is preferred to chemotherapy in advanced stage disease	13	4	8
g. It's indicated in patients undergone immunotherapy who got progression disease	88	42	19
	25	8	4
h. I don't know	0	17	31
Q22. Which follow up program do you have at your centre for MCC patients?			
a. Clinical and instrumental examinations only in suspected clinical cases			
b. Clinical and dermatological and instrumental examinations only in suspected clinical cases	6	7	0
c. Periodical first level instrumental examinations (Chest X-ray and ultrasound)	13	7	13
	25	7	0
d. Periodical second level instrumental examinations (CT scan/ FDG-PET)	56	14	47
e. I don't know	0	64	20
f. I refer the patient to other centres	0	0	20
Q23. Which are the most common side effects associated to immunotherapy? (multiple anwers)			
a. Fatigue	63		

b. Decreased appetite<br>c. Nausea, vomiting<br>d. Constipation, diarrhea<br>e. Allergic reaction to the infusion<br>f. Other	19<br>19<br>0<br>44<br>13		

Table. S3 Questionnaire items with answers (expressed as percentages) of50 Anatomo-pathologists involved in cutaneous tumor management

Q1. How old are you?	
a. <30<br>b. 30-40<br>c. 41-50<br>d. 51-60<br>e. >60	6<br>22<br>16<br>30<br>26
Q2. In which province of Lazio do you work?	
a. Roma<br>b. Latina<br>c. Fronsinone<br>d. Viterbo<br>e. Rieti	90<br>8<br>0<br>2<br>0
Q3. How much do you consider yourself updated about MCC?	
a) 0<br>b) 1<br>c) 2<br>d) 3<br>e) 4<br>f) 5<br>g) 6<br>h) 7<br>i) 8<br>j) 9<br>k) 10	0<br>6<br>6<br>8<br>22<br>18<br>14<br>10<br>6<br>8<br>2
Q4. How much importance would you give to a specific training about rare skin tumors in your everyday clinical practice?	
a. 0<br>b. 1<br>c. 2<br>d. 3<br>e. 4<br>f. 5<br>g. 6<br>h. 7<br>i. 8<br>j. 9<br>k. 10	0<br>8<br>6<br>4<br>12<br>10<br>6<br>14<br>12<br>10<br>18
Q5. How much importance would you give to a continous update about the diagnostic and therapeutic management of MCC in Lazio region?	
a. 0<br>b. 1<br>c. 2<br>d. 3<br>e. 4	0<br>6<br>6<br>0<br>10

f. 5<br>g. 6<br>h. 7<br>i. 8<br>j. 9<br>k. 10	4<br>10<br>10<br>12<br>16<br>26
Q6. In the center where you work, how many Merkel cell carcinoma patients are averagely diagnosed in a year?	
r. 1 case<br>s. Up to 3 cases<br>t. Up to 5 cases<br>u. More than 5 cases<br>v. No cases<br>w. I don't know	22<br>22<br>16<br>14<br>10<br>16
Q7. In your Center, is it planned a multidisciplinary evaluation for MCC?	
d. Yes, there is a multidisciplinary defined team<br>e. There is not a defined MDT team but specialized consultation may be requested<br>f. No, the patient was referred to another center	13<br>54<br>33
Q8. If there is the possibility of a multidisciplinary team valuation, which specialists are involved in the process? (it's possible to flag all options)	
a. Dermatologists<br>b. Plastic surgeons<br>c. Anatomopathologists<br>d. Medical oncologists<br>e. Radiaton therapists<br>f. Nuclear medicine physician<br>g. Pharmacists<br>h. General practitioners<br>i. Case manager<br>j. Psychologists<br>k. Palliative care doctors<br>l. Other	74<br>62<br>76<br>60<br>30<br>14<br>6<br>2<br>8<br>10<br>2<br>10
Q9. Would it be necessary for you/your center to add a telemedicine platform to your multidisciplinary team to improve the management of MCC patients who agree to consult other specialists or specialized teams with an important experience on MCC?	
a) Yes, always<br>b) Yes, only for complex selected cases<br>c) No, the resources are available only in presence<br>d) No, I don't consider it necessary	58<br>20<br>8<br>14
Q10. Do you think that a specialist is important for the histological diagnosis of MCC?	
a. Yes<br>b. No<br>c. Only in specific complex cases (Atypical forms or deviant immunophenotype, abnormal localization, atypical morphology, rare phenotype, combinant forms)	44<br>50<br>6
Q11. How often do you refer to a specialist a suspect case of MCC?	
a. Never<br>b. Always<br>c. Almost 25% of cases	38<br>28<br>20

d. Almost 50% of cases<br>e. Almost 75% of cases<br>f. Other	6<br>4<br>4
Q12. Has it ever happened to you that after diagnosing an MCC, the examined material was requested for further evaluation from other specialists?	
a. Never<br>b. Always<br>c. Almost 25% of cases<br>d. Almost 50% of cases<br>e. Almost 75% of cases<br>f. Other	48<br>8<br>26<br>12<br>2<br>6
Q13. How often has occurred that even in a certain MCC histological diagnosis at your work centre, the research of MPCyV in the histological material was performed?	
a. Never<br>b. Always<br>c. Almost 25% of cases<br>d. Almost 50% of cases<br>e. Almost 75% of cases<br>f. Other	68<br>10<br>10<br>0<br>2<br>10
